# Optimization of PEI-mediated transient transfection for antibody production in Expi293F cells by iron ion supplementation

**DOI:** 10.1016/j.bbrep.2026.102799

**Published:** 2026-09-21

**Authors:** Yu Liu, Shang Xiao, Yuqian Gui, Senyi Gong, Yuwen Hu, Jinzhao He, Touseef Ur Rehman, Ali Mohsin, Meijin Guo

**Affiliations:** State Key Laboratory of Bioreactor Engineering, East China University of Science and Technology, Shanghai, 200237, PR China

**Keywords:** HEK293 cells, Transient transfection, Polyethylenimine, Iron ions, Ferric ammonium citrate, Anti-PD-1 antibody

## Abstract

Polyethylenimine (PEI)-mediated transient transfection represents a pivotal methodology for recombinant protein production in HEK293 and derived cell lines. Despite extensive application, substantial potential for enhancement in transfection efficiency and exogenous protein expression persists, with the role of metal ions remaining inadequately characterized. We hypothesized that iron ions exert a concentration-dependent, biphasic regulation on PEI-mediated transfection, affecting both extracellular complex formation and intracellular gene expression. To test this hypothesis, we systematically investigated the impact of ferric ammonium citrate (FAC) on transient transfection efficiency and anti-PD-1 antibody production in Expi293F cells. Supplementation of iron-depleted basal medium with 10 μM FAC was identified as optimal, achieving 63.7% transfection efficiency for enhanced green fluorescent protein (eGFP) and an anti-PD-1 antibody yield of 55.0 mg/L. Transmission electron microscopy analysis revealed that FAC modulates transfection efficiency by altering PEI/DNA complex morphology. Quantitative real-time PCR demonstrated significant enhancement of intracellular DNA transcription by both FAC and calcium ions. The histone deacetylase inhibitor valproic acid further augmented transcription efficiency by 2.8-fold. Ultimately, anti-PD-1 monoclonal antibody production reached 171.0 mg/L in a 3-L bioreactor, representing a 10.6-fold improvement over initial conditions. This study provides preliminary evidence for establishing an efficient transient expression platform while offering initial mechanistic insights into how iron ions influence PEI-mediated transfection via complex morphology and transcriptional regulation.

## Highlights

• Ferric ammonium citrate alters polyethylenimine/DNA complex, boosting transfection.
• Iron boosts transfection by altering complex structure and promoting transcription.
• Valproic acid synergizes with iron to further increase recombinant protein yield.
• Integrated process yields 171.0 mg/L anti-PD-1 antibody in 3L bioreactor (10.6-fold).

## Graphical abstract

Protein expression pathway mediated by PEI/DNA complex. (Created by Adobe Illustrator).

**Figure:**
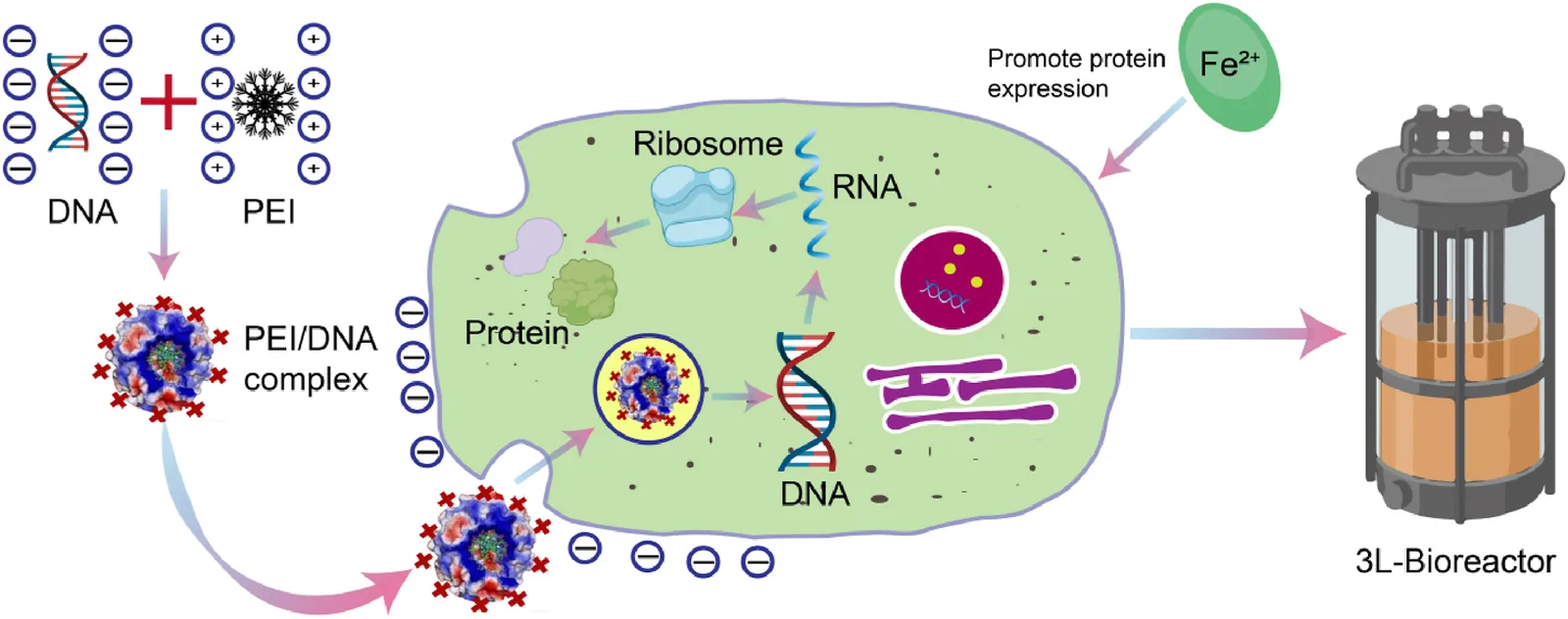

## 1 Introduction

Recombinant proteins are indispensable tools in modern biomedical research, drug discovery, diagnostics, and therapeutics, holding substantial commercial and clinical value [1,2]. Their production is facilitated by diverse exogenous expression systems, ranging from prokaryotic organisms like *E.coli* to eukaryotic systems such as yeast, insect cells, and mammalian cells [3,4]. Among these, human mammalian cell expression systems are often the platform of choice for producing complex therapeutic antibodies, enzymes, and virus-like particles [5,6]. This preference stems from their unique ability to perform sophisticated post-translational modifications, including glycosylation, phosphorylation, and proper protein folding, yielding products that are highly analogous to native human proteins in structure, function, and immunogenicity [7,8]. About the quality of SDS PAGE image the experimental repeats always similarly yielded with low quality due to the technical limitations of the gel documentation system used, herein.

The HEK293 cell line, established in the 1970s through the transformation of human embryonic kidney cells with adenovirus 5 DNA, has emerged as a particularly versatile and widely adopted host [9,10]. Its popularity is attributed to a combination of favorable characteristics: facility for both adherent and suspension culture, rapid growth kinetics, high transfection efficiency, and a proven capacity to produce proteins [9,11]. Consequently, HEK293 cells and their derived subclones are extensively utilized for the production of recombinant proteins, viral vectors for gene therapy and vaccination, and other biologics. Notably, the Expi293F cell line, a derivative adapted to high-density serum-free suspension culture, is exceptionally suitable for scalable transient protein production.

The production of recombinant proteins in mammalian cells is primarily achieved through either stable or transient expression [12]. Stable expression requires genomic integration of the foreign gene and lengthy clonal selection, often spanning several months [13]. In contrast, transient gene expression does not involve genomic integration. Instead, foreign DNA is introduced into the host cells via chemical, physical, or biological methods, leveraging the cell's native machinery for rapid protein production over a short period, typically days to two weeks [14,15]. The key advantages of this approach are its speed, flexibility, and general applicability, allowing for the production of milligram to gram quantities of protein for early-stage research and pre-clinical studies [16].

Among chemical transfection reagents, polyethylenimine (PEI) has gained prominence as a widely utilized cationic polymer [17]. PEI exists in two main architectures: linear and branched. Branched PEI contains primary, secondary, and tertiary amines, conferring a high buffering capacity that facilitates endosomal escape via the “proton sponge” effect. Linear PEI, on the other hand, consists predominantly of secondary amines and, when formulated at neutral pH, exhibits a lower charge density but often yields higher transfection efficiency with reduced cytotoxicity in many cell types [18]. Molecular weight is another critical parameter. High-molecular-weight PEI (25–800 kDa) generally provides stronger DNA condensation and higher transfection efficiency, but may also increase cytotoxicity [19]. Intermediate molecular weights around 25-40 kDa are frequently used to balance efficacy and cell viability. For transient gene expression in suspension-adapted mammalian cells, linear PEI of approximately 40 kDa has become a benchmark reagent due to its reproducible performance, serum-compatibility, and scalability [20]. Based on these considerations, we selected linear PEI of 40 kDa for this study, as it offers an optimal trade-off between transfection efficiency and cell health under serum-free conditions [21]. PEI's high amine density facilitates electrostatic interaction with negatively charged DNA phosphate backbones, condensing genetic material into stable nanoparticles. Cellular internalization occurs through endocytosis, with subsequent endosomal escape mediated by the proton sponge effect [22]. The methodology's simplicity, serum-free compatibility, and scalability establish it as a cornerstone technology for transient protein production.

Despite the maturity of PEI-based transient transfection, its efficiency and the resulting recombinant protein yields are influenced by a multitude of factors and remain subject to significant optimization. Trace elements, though required in minute quantities, play pivotal roles as enzyme cofactors in critical cellular processes, including energy metabolism, antioxidant defense, and nucleic acid synthesis [23]. While the importance of elements like selenium, zinc, and copper in cell culture has been documented, the specific role of iron ions in the context of PEI-mediated transient transfection is less understood [24]. Iron is a crucial component of hemoproteins, cytochromes, and numerous other enzymes involved in oxygen transport, electron transfer, and DNA synthesis [25,26]. While iron is often referred to as a trace element in a nutritional context, in standard cell culture media it is typically supplied at low micromolar levels and is therefore more accurately described as a supplemental metal ion. In cell culture, it is typically supplied as a transferrin-bound complex or as a chelated salt like ferric ammonium citrate (FAC) [27,28]. Some studies in stable CHO cell cultures have indicated that optimized iron supplementation can enhance specific recombinant protein yields [29,30]. However, a systematic investigation into how different chemical forms and concentrations of iron influence the efficiency of PEI-mediated transient transfection is largely absent. The mechanisms underlying any observed effects remain particularly obscure.

Therefore, we formulated the following hypothesis: iron ions exert a concentration-dependent, biphasic regulation on PEI-mediated transient transfection, at optimal concentrations, they preserve PEI/DNA complex morphology to facilitate cellular delivery, while post-internalization, they enhance transgene transcription by serving as essential cofactors for transcriptional enzymes. The main aim of this preliminary study was to systematically investigate the impact of FAC on transient transfection efficiency and recombinant protein production in Expi293F cells, and to provide an initial assessment of an optimized, scalable transient expression platform. The novelty of this work lies in three aspects: (i) it provides the first mechanistic dissection of iron's dual role in transient gene delivery; (ii) it directly links iron concentration to PEI/DNA complex morphology via TEM; and (iii) it demonstrates an unprecedented anti-PD-1 antibody titer of 171.0 mg/L in a 3-L bioreactor using an integrated optimization strategy combining FAC supplementation and valproic acid. Fig. 1 outlines the overall research workflow of this study. While preliminary in nature, these findings offer a potential protocol-driven roadmap for process intensification in biopharmaceutical development that merits further investigation.

**Fig. 1:**
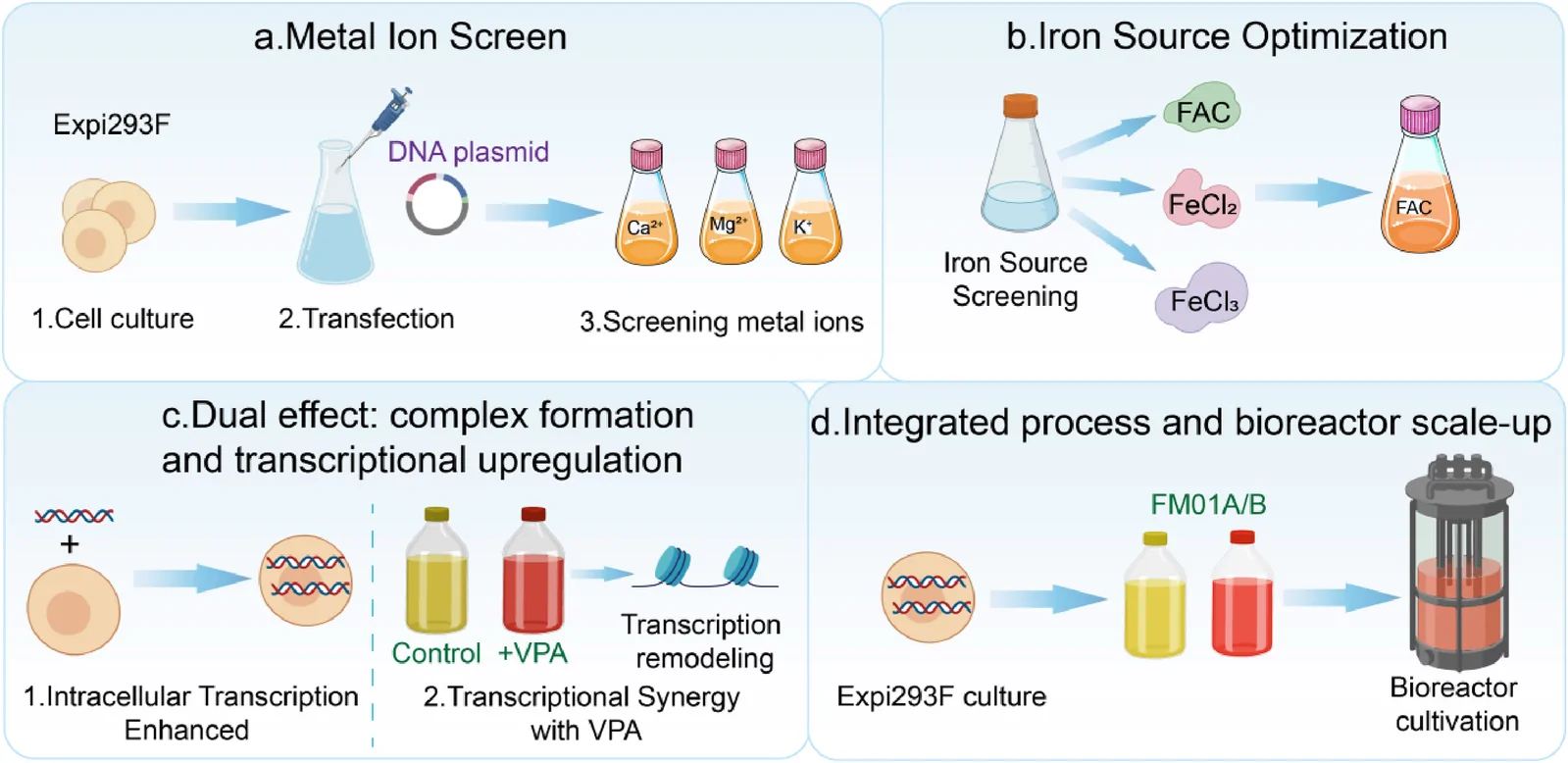
Schematic workflow of iron ion-mediated optimization of PEI transient transfection in Expi293F cells. (The figure was created using Adobe Illustrator.)

## 2 Materials and methods

### 2.1 Plasmid DNA preparation

The enhanced green fluorescent protein (eGFP) gene was cloned into a mammalian expression vector under the control of a cytomegalovirus (CMV) promoter. The anti-PD-1 monoclonal antibody heavy and light chain genes were similarly cloned into separate mammalian expression vectors, each under a CMV promoter. All plasmids were propagated in *E.coli* strain DH5α. Chemically competent DH5α cells were transformed with the respective plasmids and selected on LB agar plates containing 100 μg/mL ampicillin. A single colony was inoculated into 200 mL of LB medium supplemented with 100 μg/mL ampicillin and cultured overnight at 37°C with shaking at 220 rpm. High-purity, endotoxin-free plasmid DNA was then extracted from the bacterial pellets using an Endo-free Plasmid Maxi Kit (DP120-01, Tiangen Biotech, China), strictly according to the manufacturer's instructions. The concentration and purity of the eluted DNA were quantified using a NanoDrop 2000 spectrophotometer (Thermo Fisher Scientific, USA). Plasmid DNA aliquots were stored at −20°C until use. For anti-PD-1 antibody transfections, heavy chain and light chain plasmid solutions were mixed at a 1:1 mass ratio prior to complex formation [31].

### 2.2 Cell culture and maintenance

Expi293F cells (preserved at the State Key Laboratory of Bioreactor Engineering, East China University of Science and Technology) were maintained in suspension culture using HEK293 CD01 medium (YYCD01, Shanghai Youyi Biotechnology Co., Ltd., China), a serum-free, chemically defined medium. Cells were cultured in 125 mL, 1 L, or 5 L polycarbonate Erlenmeyer flasks (Corning, USA) with working volumes of 30 mL, 200 mL, and 1 L, respectively, which typically do not exceed 30% of the total flask volume. Cultures were incubated in a Herocell C1S incubator shaker (Shanghai Rundu Biotechnology Co., Ltd., China) set to 37°C, 5% CO_2_, 80% humidity, and an agitation speed of 120 rpm. Cell concentration and viability were monitored every 2-3 days using a Countstar Mira FL automated cell counter (Shanghai Ruiyu Biotechnology Co., Ltd., China) with the trypan blue exclusion method. For routine passage, cells were centrifuged at 100 × g for 5 min, the supernatant was discarded, and the cell pellet was resuspended in fresh pre-warmed CD01 medium to a seeding density of 3.0-5.0 × 10^5^ viable cells/mL. All manipulations were performed under aseptic conditions in a Class II A2 biological safety cabinet (Thermo Fisher Scientific, USA).

### 2.3 Transient transfection procedure

Transient transfections were performed when cultures reached a cell density of approximately 4.0 × 10^6^ viable cells/mL with a viability >95%. For transfection in shake flasks, the required volume of cell culture was centrifuged (100 × g, 5 min), and the cell pellet was resuspended in the appropriate experimental medium (CD01, CD03, or CD03 supplemented with specific additives) to achieve the target cell density for transfection. The PEI/DNA complexes were formed at a fixed PEI MAX (Linear, 40 kDa, 1 mg/mL stock solution, pH 7.0) (78pei40000, Shanghai Qifa Experimental Reagent Co., Ltd., China) to DNA ratio of 3:1 (w/w) [32]. Briefly, the required amount of DNA was diluted in a volume of the corresponding serum-free medium equal to 1/10th of the final transfection volume (1 mL for a 10 mL final volume in a 125 mL flask). In a separate tube, the appropriate amount of PEI stock solution was diluted in an equal volume of the same serum-free medium. The PEI solution was then added dropwise to the DNA solution under gentle vortexing [33]. The mixture was incubated at room temperature for 25 min to allow for complex formation. After the incubation period, the PEI/DNA complexes were added dropwise to the cell suspension while gently swirling the flask. The transfected culture was then returned to the incubator shaker.

### 2.4 Bioreactor scale-up culture

A 3L benchtop bioreactor (Shanghai Senson Biotechnology Co., Ltd., China) with a working volume of 1 L was used for scale-up studies. The bioreactor was equipped with pH and dissolved oxygen (DO) probes (Hamilton, Switzerland). The vessel, containing the basal medium (CD03 supplemented with 10 μM FAC), was sterilized *in situ* by autoclaving at 121°C for 30 min. The pH probe was calibrated offline before sterilization using standard buffers (pH 4.01 and 6.86). Post-sterilization, the DO probe was calibrated to 0% and 100%. The bioreactor was inoculated with Expi293F cells at a density of 1.0 × 10^6^ cells/mL. The culture conditions were set and controlled as follows: temperature, 36.5°C; pH, 7.0 ± 0.25; DO, 40% ± 5%. Antifoam B (A5757, Sigma-Aldrich, USA) was added manually as needed to control foam. The transfection was performed *in situ* at a cell density of 4.0 × 10^6^ cells/mL using the optimized protocol. The feeding strategy involved the addition of 5% (v/v) FM01A (YYFM01A, Shanghai Youyi Biotechnology, China) and 0.5% (v/v) FM01B (YYFM01B, Shanghai Youyi Biotechnology, China) starting at 24 h post-transfection and then every other day. Glucose was supplemented daily from day 2 onwards based on offline measurements to maintain a concentration above 6.0 g/L.

### 2.5 Metal ion and additive studies

The iron-free basal medium, HEK293 CD03 (YYCD03, Shanghai Youyi Biotechnology, China), was used as the base for all metal ion and additive studies to allow precise control over supplementation. For the initial metal ion screen, KCl (P5405, Sigma-Aldrich, USA) (1.34 mM), CuSO_4_ (C1297, Sigma-Aldrich, USA) (3.1 μM), MgCl_2_ (63069, Sigma-Aldrich, USA) (1.05 mM), CaCl_2_ (C5670, Sigma-Aldrich, USA) (90 μM), NaCl (S9888, Sigma-Aldrich, USA) (8.6 mM), ZnSO_4_ (Z2876, Sigma-Aldrich, USA) (31 μM), AlCl_3_ (563919, Sigma-Aldrich, USA) (7.5 μM), CoCl_3_ (361844, Sigma-Aldrich, USA) (30 μM), and FAC (40 μM) (F5879, Sigma-Aldrich, USA) were individually added to CD03 medium from filter-sterilized stock solutions. The additive concentrations were determined based on formulations commonly used in existing culture media. For iron optimization, FAC, FeCl_3_, and FeCl_2_ were tested at concentrations of 5, 10, 20, 40, and 80 μM in CD03 medium. Valproic acid (VPA, PHR1061, Sigma-Aldrich, USA) was prepared as a 500 mM stock solution in dimethyl sulfoxide (DMSO) and was added to the FM01A feed medium to achieve final concentrations of 3.0 mM upon its addition to the culture. The culture media and additives used in this experiment are summarized in Table 1.

**Table 1:** Experimental design for process optimization in Expi293F transient transfection.

Experimental Phase	Basal Medium	Supplement	Supplementation Strategy	Corresponding Figure	Purpose
Phase I: Metal Ion Screening	HEK293 CD01 (iron-containing)	None	None	Fig. 2A–B	Baseline efficiency in standard medium.
	HEK293 CD03 (iron-free)	1.34 mM KCl, 3.1 μM CuSO_4_, 1.05 mM MgCl_2_, 90 μM CaCl_2_, 8.6 mM NaCl, 31 μM ZnSO_4_, 7.5 μM AlCl_3_, 30 μM CoCl_3_, 40 μM FAC	None		Screening for specific metal ion effects on transfection.
Phase II: Iron Source and Concentration Optimization	HEK293 CD03 (iron-free)	5, 10, 20, 40, 80 μM FAC	None	Fig. 3, Fig. 4, Fig. 5	Determining optimal iron source and concentration without cytotoxicity.
		10, 20, 40, 80 μM FeCl_2_	None		
		10, 20, 40, 80 μM FeCl_3_	None		
Phase III: Enhanced Production	HEK293 CD03 (iron-free)	10 μM FAC	None	Fig. 6	Validating the optimal condition for anti-PD-1 mAb yield (55.0 mg/L).
		10 μM FAC + 3 mM VPA	FM01A (5% v/v) + FM01B (0.5% v/v)	Fig. 10, Fig. 11	Synergistic effect of VPA on transcription and ultimate high-yield production.

### 2.6 Analytical methods

Cell Density and Viability Monitoring: Cell concentration and viability were routinely monitored using a Countstar Mira FL automated cell counter (Shanghai Ruiyu Biotechnology Co., Ltd., China) based on the trypan blue exclusion method. Briefly, 200 μL of cell suspension was mixed with an equal volume of 0.2% (w/v) trypan blue solution. A 20 μL aliquot of the mixture was then loaded onto a disposable counting slide and analyzed by the instrument. The Countstar Mira FL automatically calculates total cell density (viable cells/mL) and the percentage of viable cells (unstained) versus dead cells (stained blue) based on built-in image analysis algorithms. All measurements were performed in at least three independent biological replicates, and data are presented as the mean ± standard deviation.

Transfection Efficiency Analysis via Flow Cytometry: At 48 h post-transfection with the eGFP plasmid, 200 μL of cell suspension was collected and centrifuged at 300 × g for 5 min. The cell pellet was washed twice with 500 μL of ice-cold PBS and resuspended in 300 μL of PBS containing 1% (w/v) bovine serum albumin (BSA, ST023, Beyotime, China). The cell suspension was filtered through a 35 μm nylon mesh to remove aggregates before analysis. Gating strategy: The viable cell population was first identified on a forward scatter (FSC-A) versus side scatter (SSC-A) dot plot to exclude debris and aggregates, as these non-cellular events typically exhibit low FSC and SSC signals. Subsequently, doublets were excluded using FSC-H versus FSC-A gating to ensure that only single cells were analyzed. Transfection efficiency was then determined as the percentage of eGFP-positive cells within this viable, single-cell gate, with the threshold for positivity set using untransfected control cells (negative control). EGFP fluorescence was measured using a CytoFLEX LX flow cytometer (Beckman Coulter, USA) with an excitation wavelength of 488 nm and emission detection in the FITC channel. For each sample, at least 10,000 events were collected. Data analysis was performed using CytExpert software (Beckman Coulter). The transfection efficiency was calculated as the percentage of eGFP-positive cells in the viable cell population, gated based on forward and side scatter properties, compared to untransfected control cells.

Protein Quantification and Analysis: eGFP Expression (SDS-PAGE): For eGFP-expressing cultures, 500 μL of cell suspension was collected and centrifuged at 12,000 × g for 5 min. The cell pellet was resuspended in 100 μL of PBS and subjected to ultrasonic cell disruption on ice (2s ON, 5s OFF, 5 min total duration) using a JX-650 ultrasonic processor (Shanghai Jingxin Industrial Development Co., Ltd., China) [34]. The lysate was centrifuged at 12,000 × g for 10 min at 4°C, and 20 μL of the supernatant was mixed with 5 μL of 5× SDS-PAGE loading buffer (P0015L, Beyotime, China). The mixture was heated at 95°C for 10 min, centrifuged briefly, and 20 μL was loaded onto a 12.5% polyacrylamide gel prepared using a PAGE Gel Fast Preparation Kit (P0901S, Beyotime, China). Electrophoresis was performed at 120 V for approximately 40 min in Tris-Glycine-SDS running buffer. Gels were stained with Coomassie Brilliant Blue R-250 (C6233, Beyotime, China) and destained until clear protein bands were visible against a low-background gel. The original images, sample information, and detailed labeling of SDSPAGE in the text are shown in Figs. S1 and S2.

Anti-PD-1 Antibody Titer Measurement: For antibody-producing cultures, 300 μL of cell culture was centrifuged at 1000 × g for 10 min to remove cells and debris. The clarified supernatant was analyzed directly for antibody concentration using a Cedex Bio HT analyzer (Roche Diagnostics, Switzerland) with a proprietary immunoassay cartridge for human IgG quantification, following the manufacturer's protocol.

Antibody Purification and Quantification: In the field of antibody production, protein A affinity chromatography is a gold-standard method for the purification and quantification of monoclonal antibodies [[35], [36], [37]]. For purified yield determination, larger volumes (1 L) of culture supernatant were harvested by centrifugation at 8000 × g for 20 min at 4°C, followed by filtration through a 0.22 μm PES membrane. The antibody was purified using an ÄKTA pure 25 chromatography system (Cytiva, USA) equipped with a 1 mL Protein A affinity column (AC-ProC-1.0-800, Shanghai Yurou Biotechnology, China) [38]. The column was equilibrated with 10 column volumes (CV) of equilibration buffer (20 mM Sodium Phosphate, 150 mM NaCl, pH 7.4). The filtered supernatant was loaded at a flow rate of 1 mL/min. The column was washed with 10-15 CV of equilibration buffer until the UV absorbance (280 nm) baseline stabilized. Bound antibody was eluted with 10 CV of 100 mM Glycine-HCl buffer (pH 3.0), and the eluate was immediately neutralized with 1/10th volume of 1 M Tris-HCl buffer (pH 9.0). The protein concentration in the pooled elution fractions was determined by measuring the absorbance at 280 nm using the NanoDrop 2000 spectrophotometer, and the total yield was calculated.

### 2.7 PEI/DNA complex characterization by transmission electron microscopy (TEM)

PEI/DNA complexes were prepared at the standard 3:1 ratio in CD03 medium containing 0, 10, or 80 μM FAC, as described in section 2.3, and incubated for 25 min. A 10 μL aliquot of the complex suspension was applied onto a carbon-coated copper grid and allowed to adsorb for 20 min at room temperature. Excess liquid was carefully removed by blotting with filter paper. The grid was then negatively stained by applying a 10 μL droplet of 2% (w/v) phosphotungstic acid (pH 7.0, P4006, Sigma-Aldrich, USA) for 20 s [39]. The stain was blotted away, and the grid was rinsed briefly with a droplet of ultrapure water, which was also blotted off. This washing step was repeated twice. The grid was air-dried completely before imaging. Samples were observed using a JEM-1400 TEM (JEOL, Japan) operated at 80 kV. Of note, due to the technical limitations of the TEM instrument, the resolution of the acquired images is constrained. Nevertheless, the morphological differences described were consistently reproduced across at least three independent experiments.

### 2.8 Analysis of intracellular DNA transcription by qPCR

Cells were transfected with the eGFP plasmid under different culture medium conditions as specified in the results. At designated time points (2, 12, 24, 48 h post-transfection), approximately 1 × 10^6^ cells were collected by centrifugation (300 × g, 5 min). Total RNA was extracted from the cell pellet using a Total RNA Isolation Kit (RC201, Vazyme, China), which included a DNase I digestion step to remove genomic DNA contamination. RNA concentration and purity were assessed using the NanoDrop 2000. First-strand cDNA was synthesized from 1 μg of total RNA using a HiScript II Q RT SuperMix for qPCR (R222-01, Vazyme, China), following the manufacturer's instructions. Quantitative PCR was performed on a QuantStudio 5 Real-Time PCR System (Thermo, USA) using ChamQ Universal SYBR qPCR Master Mix (Q711-02/03, Vazyme, China) [40]. The 20 μL reaction mixture contained 10 μL of 2× Master Mix, 0.4 μL of each primer (10 μM), 2 μL of cDNA template, and 7.2 μL of nuclease-free water. The primer sequences used were shown in Table S1. The amplification protocol was: initial denaturation at 95°C for 30 s; followed by 40 cycles of 95°C for 10 s and 60°C for 30 s. The 60°C step serves for both annealing and extension, as recommended by the kit manufacturer [41,42]. A melt curve analysis was performed at the end of each run to confirm amplification specificity. The housekeeping gene GAPDH was used for normalization. The relative mRNA expression levels of eGFP were calculated using the comparative 2^(-ΔΔCt) method.

### 2.9 Data and statistical analysis

All experiments were performed in at least three independent biological replicates. Data are presented as the mean ± standard deviation. For multi-group comparisons, one-way analysis of variance (ANOVA) followed by Tukey's post-hoc test was applied. A p-value of less than 0.05 was considered statistically significant. Graphs were generated using GraphPad Prism 9 (GraphPad Software, USA).

## 3 Results

### 3.1 Comprehensive screening reveals divergent effects of metal ions on transfection

To investigate which metal ions promote transfection, 9 different metal ions were added to standard CD01 medium and their effects on eGFP transfection efficiency and cell growth were evaluated. The results in Fig. 2A shows that Calcium ions (CaCl_2_) exhibited the most pronounced positive effect; the addition of 90 μM CaCl_2_ boosted transfection efficiency dramatically to 65.4%, far exceeding the blank control group. This indicated that Ca^2+^ likely plays a facilitative role in certain steps, such as membrane stability, endocytosis, or intracellular signaling.

Conversely, iron ions in the form of FAC demonstrated a strikingly different effect. The addition of 40 μM FAC resulted in a clear inhibition of transfection efficiency (Fig. 2A), despite exhibiting a slight promotive effect on cell growth and viability per se (Fig. 2B). This phenomenon of “promoting growth but inhibiting transfection” suggested that the impact of iron ions on the transfection process is decoupled from their effect on basic cellular metabolism, implying the existence of a specific mechanism affecting the gene delivery pathway. Copper ions (CuSO_4_) displayed potent cytotoxicity, significantly reducing cell viability, consistent with their known ability to generate reactive oxygen species at elevated concentrations. The other tested ions (K, Mg, Na, Zn, Al, Co) showed negligible effects on transfection efficiency and cell status at the concentrations used. Given the central role of iron in cellular physiology and the unique inhibitory effect observed with FAC, a more detailed investigation was warranted.

**Fig. 2:**
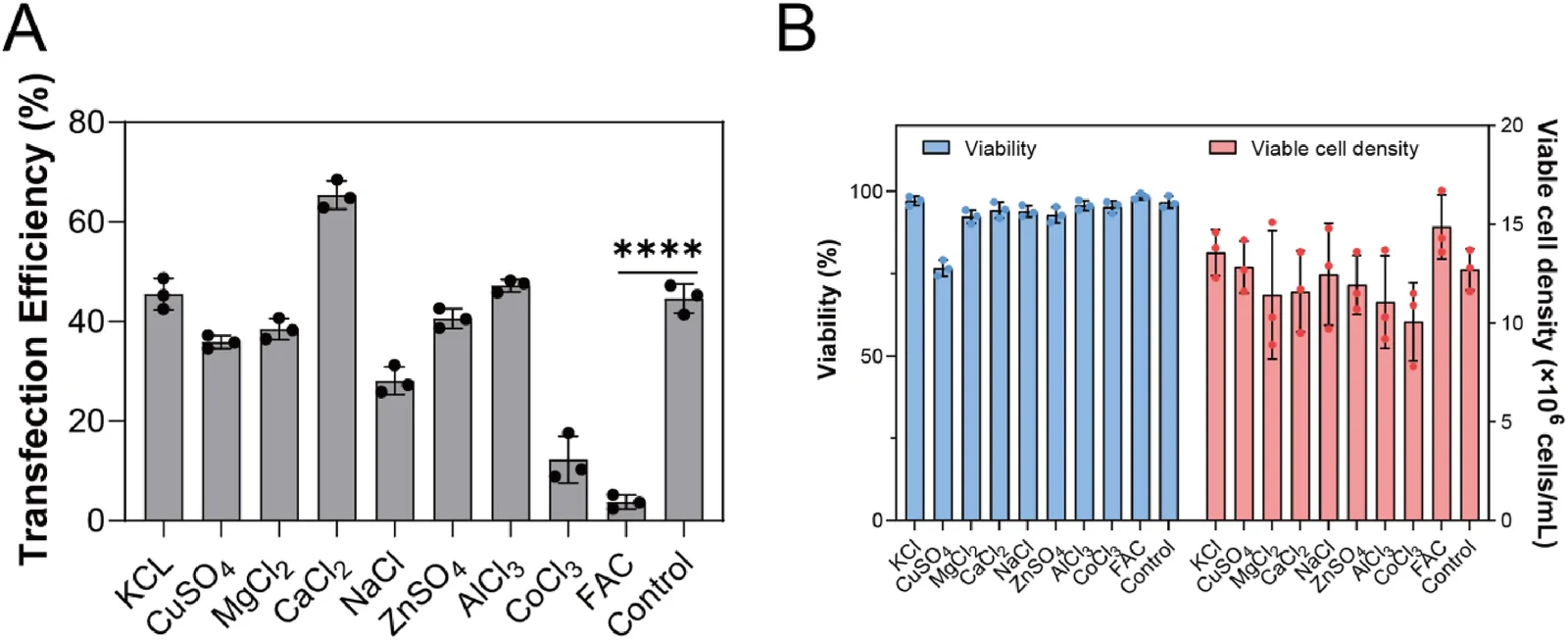
The impact of various metal ions on the transfection efficiency and cell growth of Expi293F cells transfected with the eGFP plasmid. (A) Transient transfection efficiency; (B) Viable cell density (right axis, ×10^6^ cells/mL) and viability (left axis, %) in Expi293F cells was analyzed via trypan blue exclusion method post-transfection with the eGFP plasmid. Ca^2+^ promotes efficiency (65.4%), while FAC inhibits it despite supporting growth. Data are presented as mean ± SD from three independent biological replicates (n = 3). Statistical significance was set at p < 0.05.

### 3.2 Concentration and source of iron ions critically determine transfection outcome

To precisely control the iron environment and facilitate an in-depth study, the iron-free CD03 basal medium was adopted. Three different chemical forms of iron—FAC, FeCl_3_, and FeCl_2_ were compared across a concentration gradient of 10 to 80 μM.

The experimental results revealed a consistent and unambiguous trend (Fig. 3A–C): for all three iron sources, transfection efficiency decreased monotonically as the iron ion concentration increased. The highest transfection efficiencies for each source were observed at the lowest tested concentration of 10 μM, with FAC proving most effective, elevating eGFP transfection efficiency to 63.7%, significantly higher than the efficiency achieved in the iron-containing CD01 medium (39.3%). The optimal efficiencies for FeCl_3_ and FeCl_2_ at 10 μM were 62.7% and 53.7%, respectively. It is noteworthy that across the 10-80 μM concentration range, none of the three iron sources exerted a significant negative impact on cell viability or growth density (Fig. 3D–F), indicating that the observed decline in transfection efficiency was not due to general cytotoxicity.

**Fig. 3:**
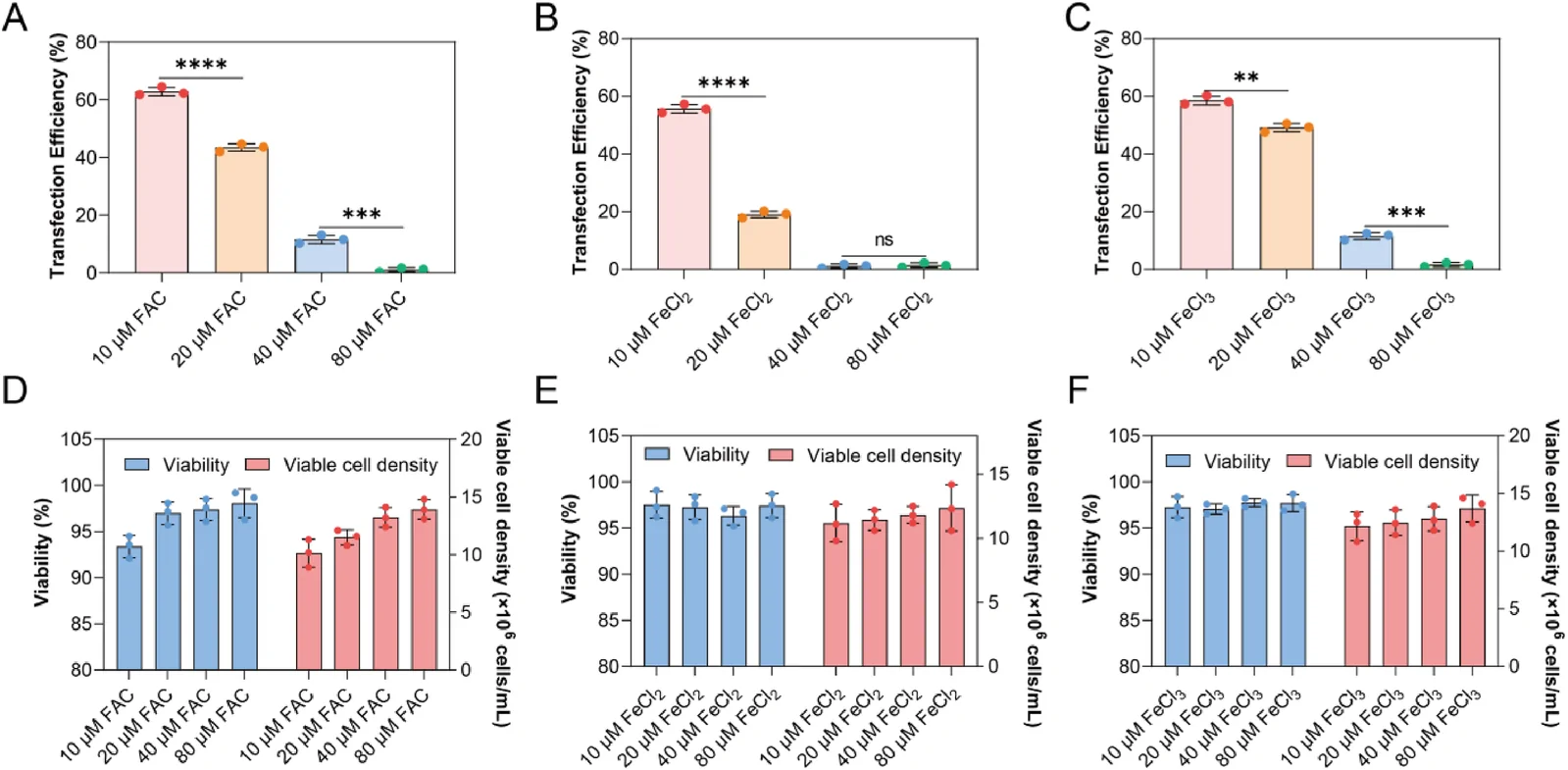
Transfection efficiency and cell growth of Expi293F cells transfected by eGFP plasmid under different ferron ions. (A-C) Transfection efficiency, (D-F) Cell viability and density. (A,D) FAC added in CD03 medium. (B,E) FeCl_2_ added in CD03 medium. (C,F) FeCl_3_ added in CD03 medium. 10 μM FAC gives the highest efficiency (63.7%). Viable cell density (right axis, ×10^6^ cells/mL) and viability (left axis, %) in Expi293F cells was analyzed via trypan blue exclusion method. Cell counting was performed using the trypan blue staining method. Data are presented as mean ± SD from three independent biological replicates (n = 3). Statistical significance was set at p < 0.05.

The effect of 5 μM FAC was further tested and found to be inferior (51.3% efficiency) to the 10 μM condition (Fig. 5A). Flow cytometry dot plots (Fig. 4A–G) provided a visual confirmation, showing the highest proportion of eGFP-positive cells under the 10 μM FAC condition. SDS-PAGE analysis (Fig. 5B) also corroborated that the eGFP protein band was most intense in the 10 μM FAC group. Meanwhile, the presence of FAC showed no significant impact on cell viability and density, demonstrating its safety profile (Fig. 5C–D). Collectively, these data established 10 μM FAC in CD03 medium as the optimal condition for transfection efficiency.

**Fig. 4:**
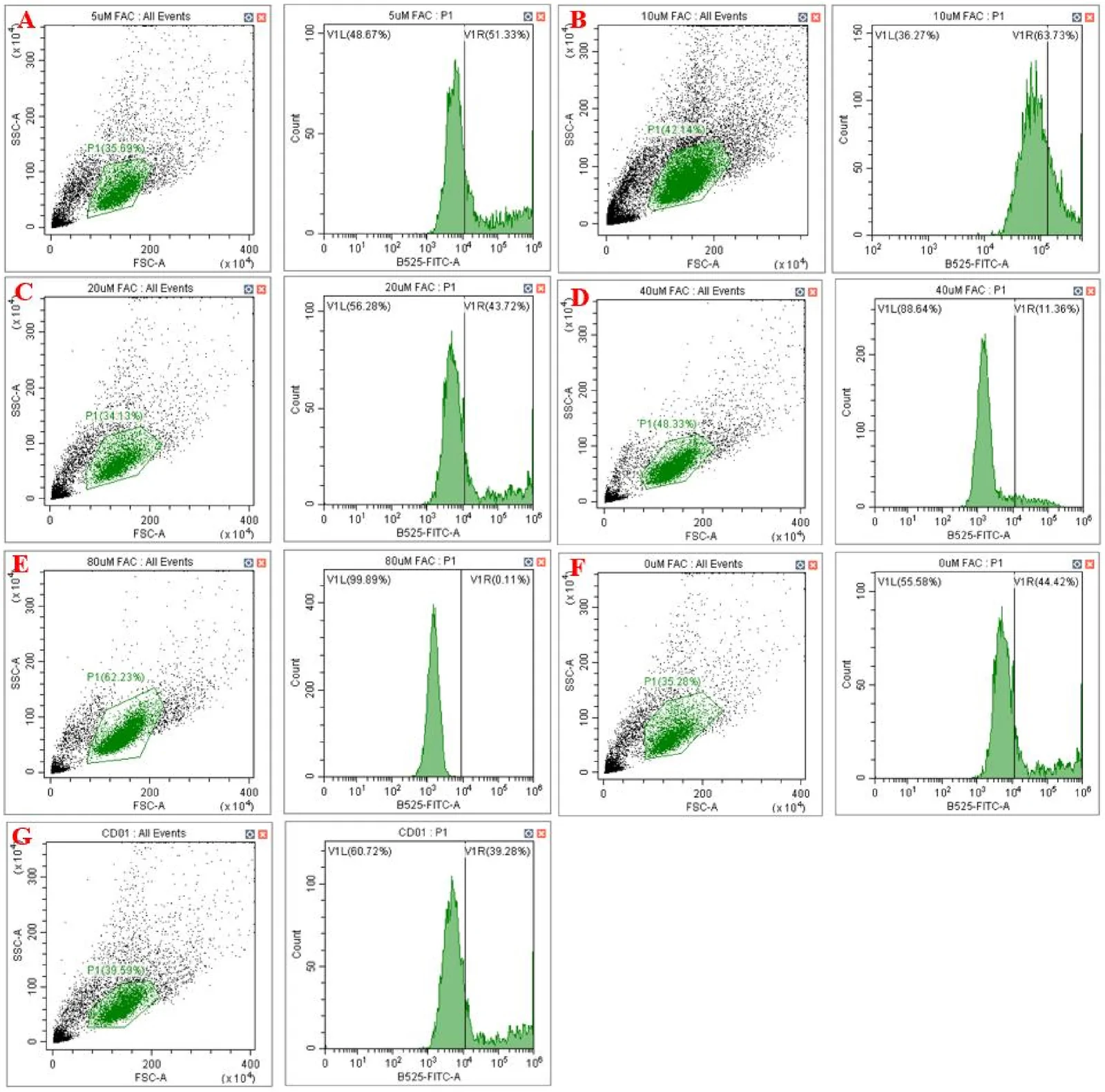
Flow cytometry of Expi293 cells transfected by eGFP plasmid under different FAC concentrations. Flow cytometry after adding (A) 5 μM FAC; (B) 10 μM FAC; (C) 20 μM FAC; (D) 40 μM FAC; (E) 80 μM FAC; (F) 0 μM FAC in CD03 medium; (G) Flow cytometry under CD01 medium. 10 μM FAC yields the highest proportion of positive cells.

**Fig. 5:**
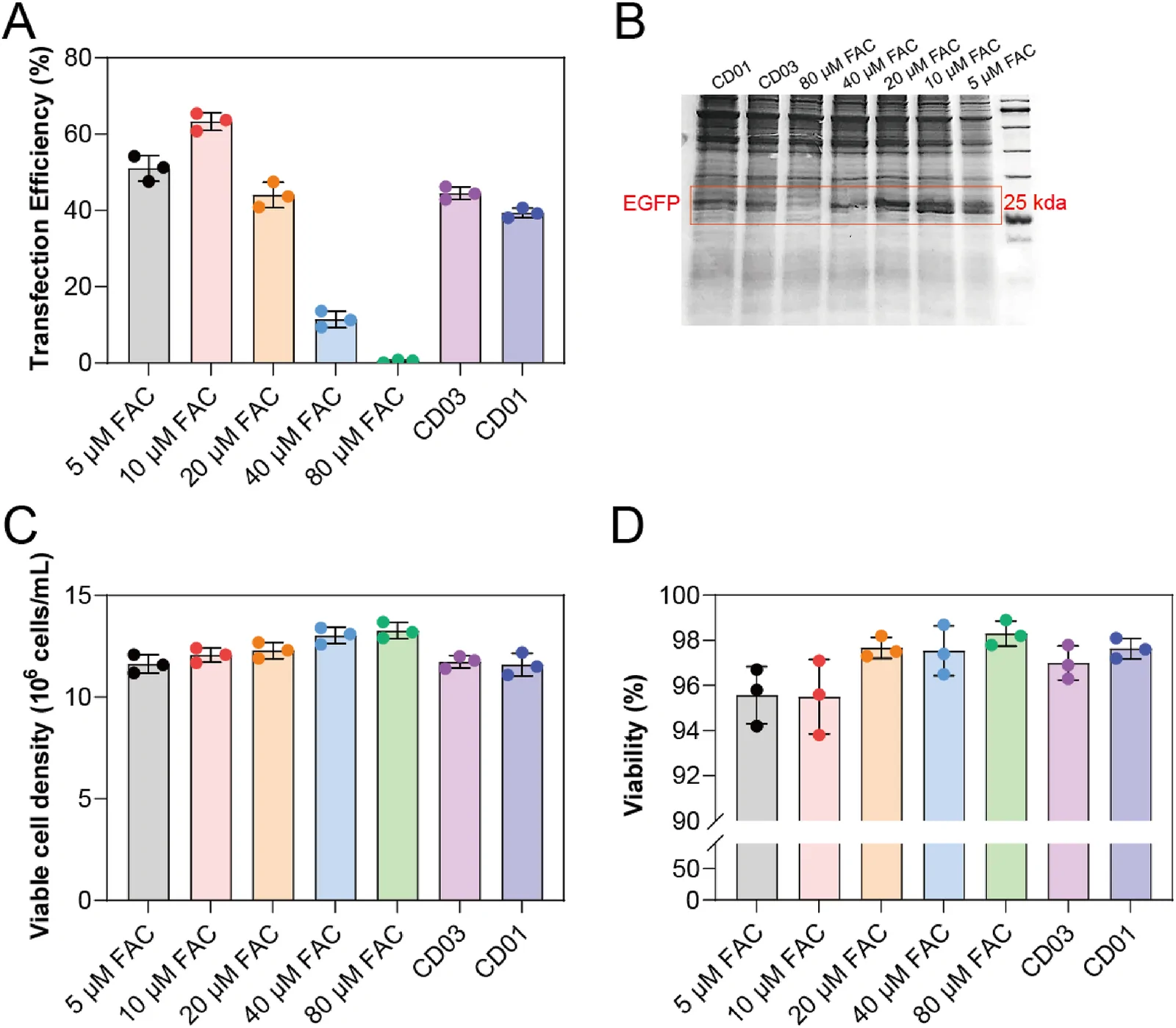
Effect of FAC concentration on eGFP expression and cell growth in transiently transfected Expi293F cells. (A) Transfection efficiency analyzed by flow cytometry. (B) SDS-PAGE analysis of eGFP protein expression in cell lysates. (C) Viable cell density and (D) cell viability post-transfection under varying FAC concentrations. Error bars represent mean ± SD of triplicate experiments. FAC up to 80 μM does not affect cell health. Cell counting was performed using the trypan blue staining method.

To confirm the universality of this optimized condition for a therapeutically relevant protein, a validation experiment was performed using the anti-PD-1 antibody plasmids. The results (Fig. 6A) were highly consistent with the eGFP experiments: the highest anti-PD-1 antibody yield, reaching 55.0 mg/L, was achieved in CD03 medium supplemented with 10 μM FAC, which was 3.2 times the yield obtained in the original CD01 medium (17.0 mg/L). SDS-PAGE electrophoresis under non-reducing conditions (Fig. 6B) showed the widest and most intense band corresponding to the intact antibody in the 10 μM FAC group. Additionally, the cells exhibited favorable growth and proliferation kinetics (Fig. 6C–D). These results unequivocally demonstrated that a completely iron-free environment is suboptimal, and supplementation with an appropriate amount of FAC is necessary for achieving high-level recombinant protein expression, whereas an excess is decidedly detrimental.

**Fig. 6:**
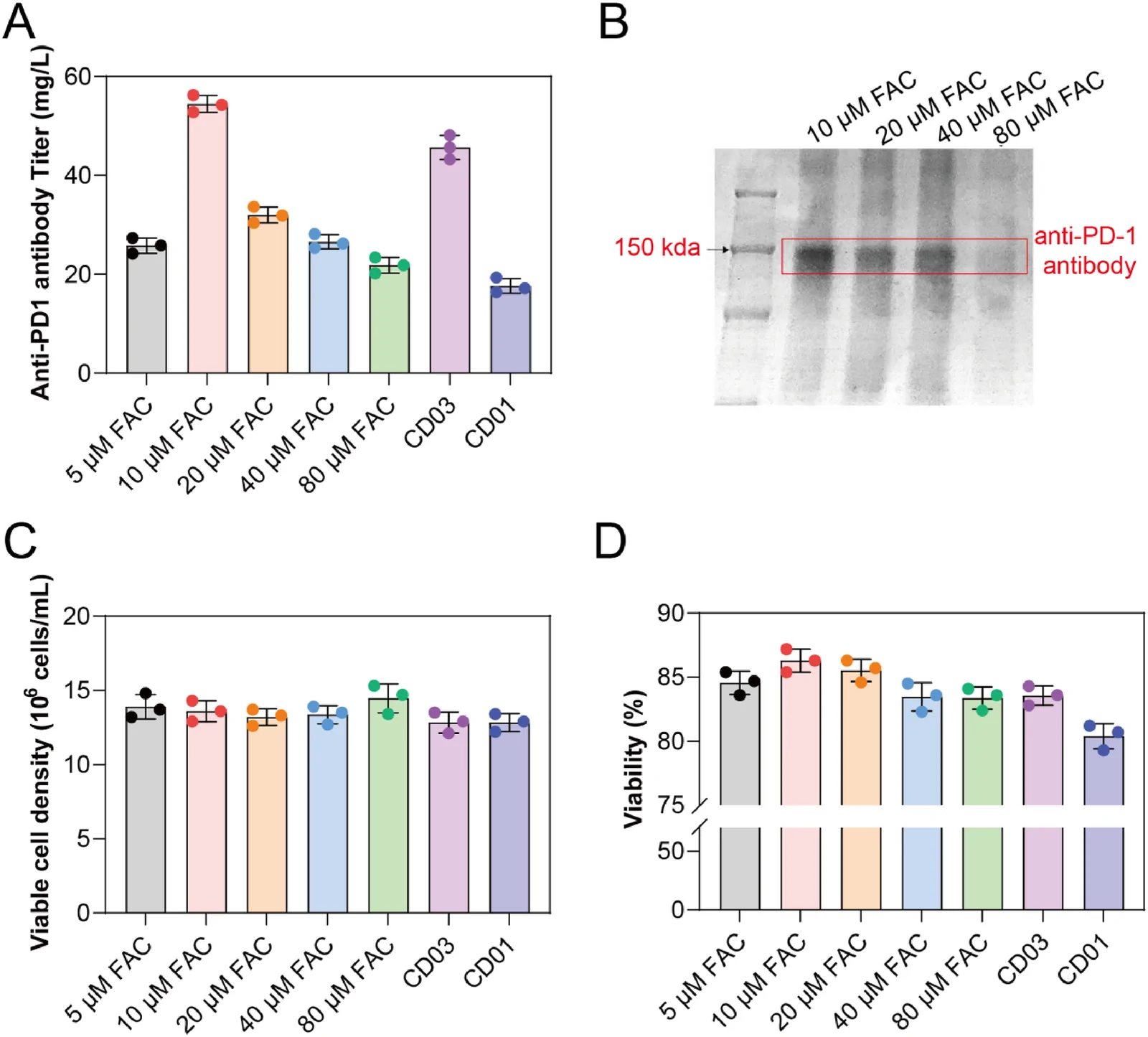
Impact of FAC concentration on anti-PD-1 antibody expression and growth of transiently transfected Expi293F cells. (A) Anti-PD-1 antibody titer measured by ELISA. (B) SDS-PAGE analysis of anti-PD-1 antibody under different conditions. (C) Viable cell density and (D) cell viability following transfection with the anti-PD-1 expression plasmid. Error bars indicate mean ± standard deviation (n = 3). Cell counting was performed using the trypan blue staining method.

### 3.3 Iron ions modulate transfection efficiency by altering PEI/DNA complex physicochemistry

Moreover, cells were incubated and transiently transfected while transfection efficiency was recorded at 48h to investigate the influence of metal ions on PEI/DNA complex formation during transient transfection. As shown in Fig. 7A, transfection efficiency decreased significantly with increasing FAC concentration. Consistent with previous observations, FAC did not compromise cell viability (Fig. 7B), confirming that the reduced efficiency was not due to toxic effects. These findings warrant further investigation into the underlying mechanism by which FAC addition impairs transfection efficiency.

**Fig. 7:**
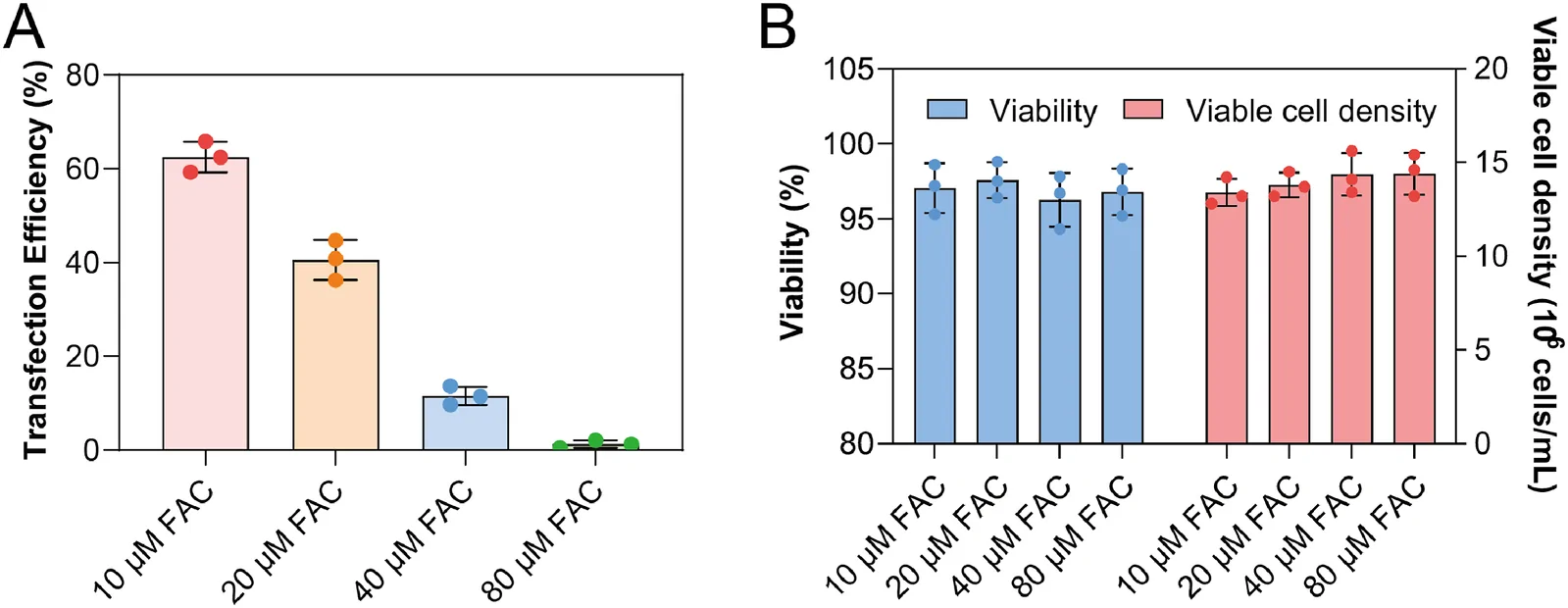
Transient transfection efficiency and post-transfection growth of Expi293F cells mediated by eGFP plasmid under different incubation conditions. (A) Transfection efficiency under different concentrations of FAC. (B) Cell viability and density under different concentrations of FAC. Cell counting was performed using the trypan blue staining method.

To unravel the physical mechanism underlying the iron concentration-dependent effect on transfection efficiency, the morphology of PEI/DNA complexes formed under different FAC concentrations was directly visualized using TEM. The TEM results provided compelling visual evidence. Complexes formed in CD03 medium devoid of FAC (Fig. 8A–C) were structurally compact, relatively homogeneous in morphology, and small in size, distributed between 0.22 and 0.29 μm. This tight structure is likely favorable for cellular uptake.

**Fig. 8:**
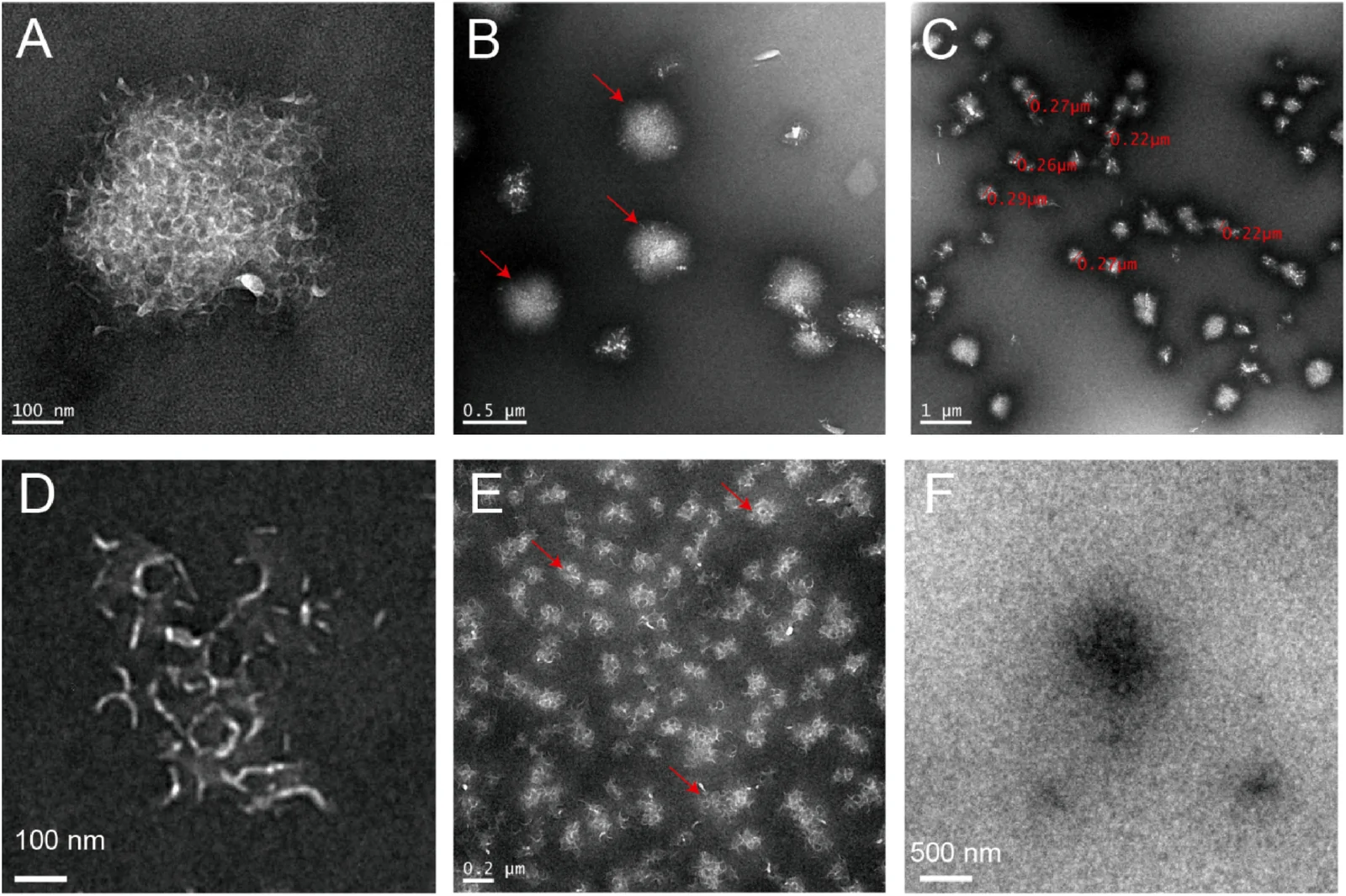
TEM images of PEI/DNA complex under different incubation conditions PEI/DNA complex state incubated in CD03 medium with (A-C) 0 μM FAC; (D-E) 10 μM FAC; (F) 80 μM FAC. Optimal efficiency correlates with 10 μM FAC structure.

Following incubation in medium supplemented with 10 μM FAC, the complex morphology underwent a remarkable transformation, adopting a unique snowflake-like or reticulated structure (Fig. 8D–E). Their particle size slightly increased, with a broader distribution range (0.27-0.54 μm). It is hypothesized that the citrate anions and iron cations from FAC may engage in subtle interactions with PEI and DNA, interfering with the complete condensation of DNA by PEI and resulting in the formation of a looser, more open structure. This architecture might be more prone to disassembly at downstream stages, such as during endosomal escape, thereby facilitating DNA release and explaining the peak transfection efficiency observed at this concentration.

However, when the FAC concentration was elevated to 80 μM, the complex morphology suffered a catastrophic change (Fig. 8F). The complexes became extremely loose, heterogeneous, and exhibited an exceptionally wide particle size distribution (0.15-0.80 μm), indicating that excessive FAC severely compromised the ability of PEI and DNA to form stable, structured complexes. These unstable, heterogeneous aggregates are likely inefficiently internalized by cells, or, even if internalized, fail to adequately protect and release DNA into the cytoplasm.

These morphological observations correlated perfectly with the transfection efficiency results (Fig. 9). They clearly demonstrate that FAC modulates transfection efficiency by directly altering the physical morphology and stability of PEI/DNA complexes. The optimal transfection efficiency corresponds to the formation of complexes with appropriate size and structure, while excessive FAC induced structural breakdown directly leads to transfection failure and a decrease in antibody expression levels.

**Fig. 9:**
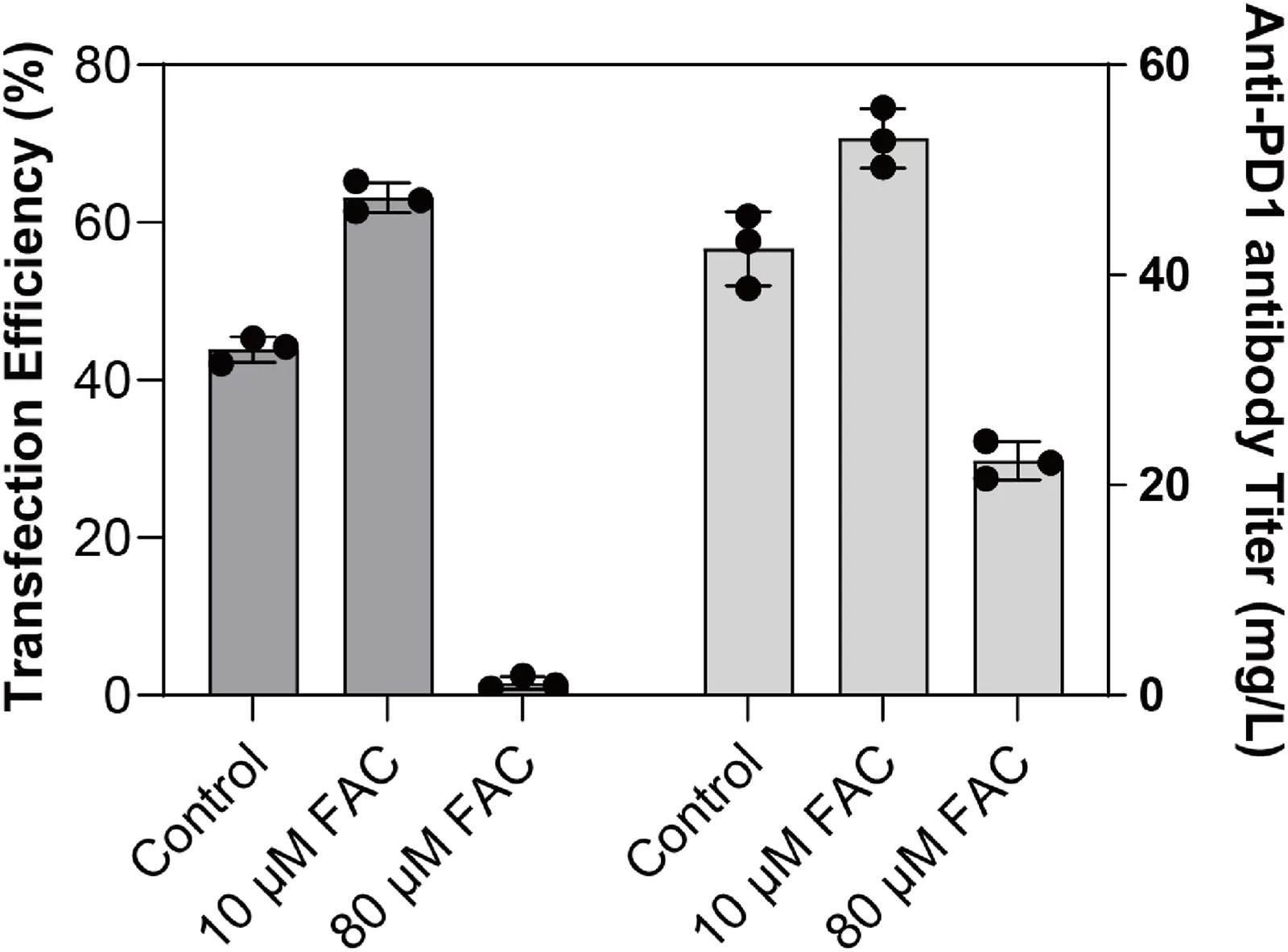
Transient efficiency and protein expression under different concentrations of FAC.

### 3.4 Metal ions and VPA potently enhance transcription of delivered DNA

While high concentrations of FAC were inhibitory during the complex formation stage, the role of iron ions after DNA delivery, during the expression phase, was investigated separately. Using qPCR, the intracellular mRNA levels of eGFP were measured in cells cultured under different medium conditions.

The results (Fig. 10A) revealed a story starkly different from the previous one. In the expression medium, the addition of FAC significantly promoted the transcription of the delivered DNA. Compared to the FAC-free CD03 group (Control), the relative intracellular eGFP mRNA content was significantly higher in groups supplemented with 10 μM and 80 μM FAC, with the 80 μM group showing higher levels than the 10 μM group. Intracellular eGFP mRNA levels were significantly higher in FAC-supplemented groups compared to the FAC-free control. This promotive effect during the expression stage stands in sharp contrast to its inhibitory effect during the delivery stage at high concentrations, explaining why the final optimal FAC concentration represents a balance point. The qPCR data also confirmed that Ca^2+^ promotes transcription, aligning with its overall efficiency enhancement.

**Fig. 10:**
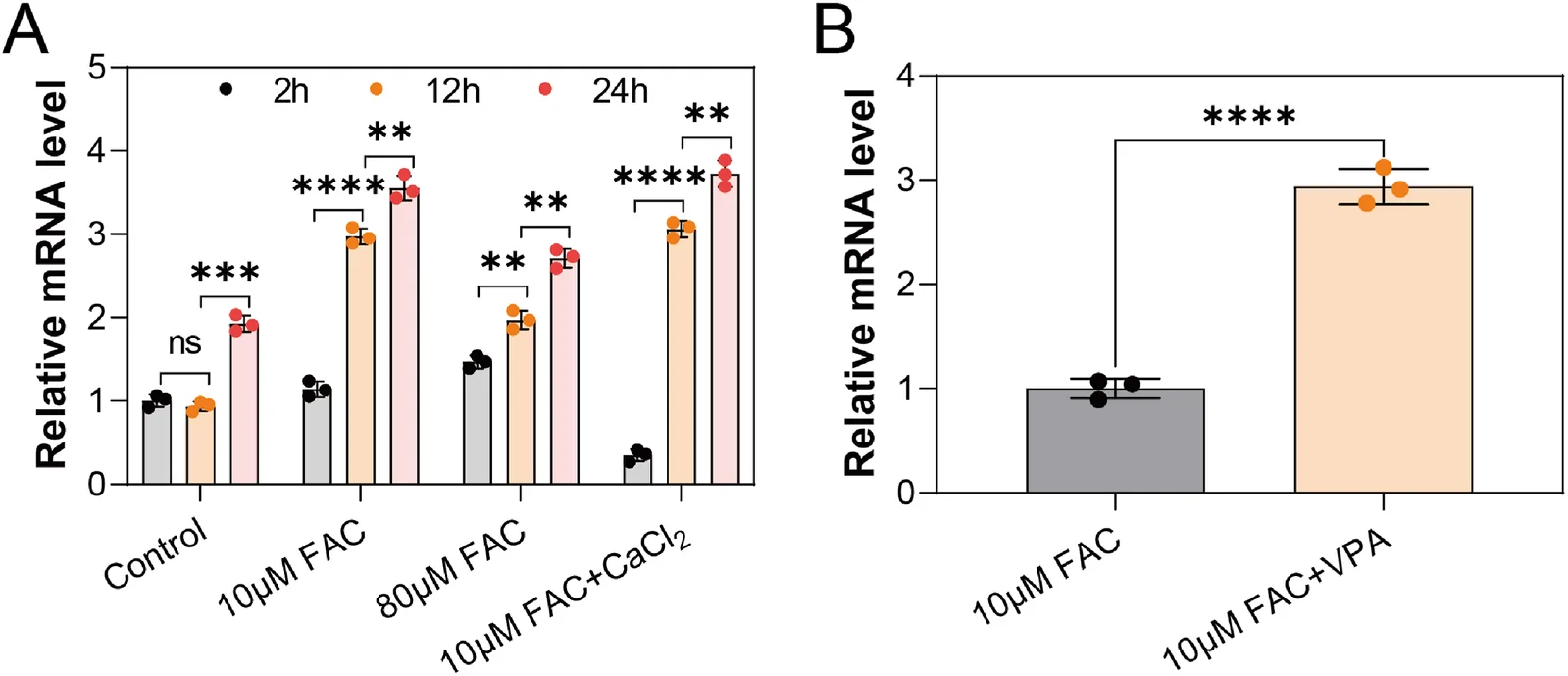
Cellular uptake of eGFP plasmid by Expi293F cells under different conditions. (A) Intake levels under different conditions at different times (2h, 4h, 12h); (B) Intake levels under different conditions after 48h. Data are presented as mean ± SD from three independent biological replicates (n = 3). Statistical significance was set at p < 0.05.

**Fig. 11:**
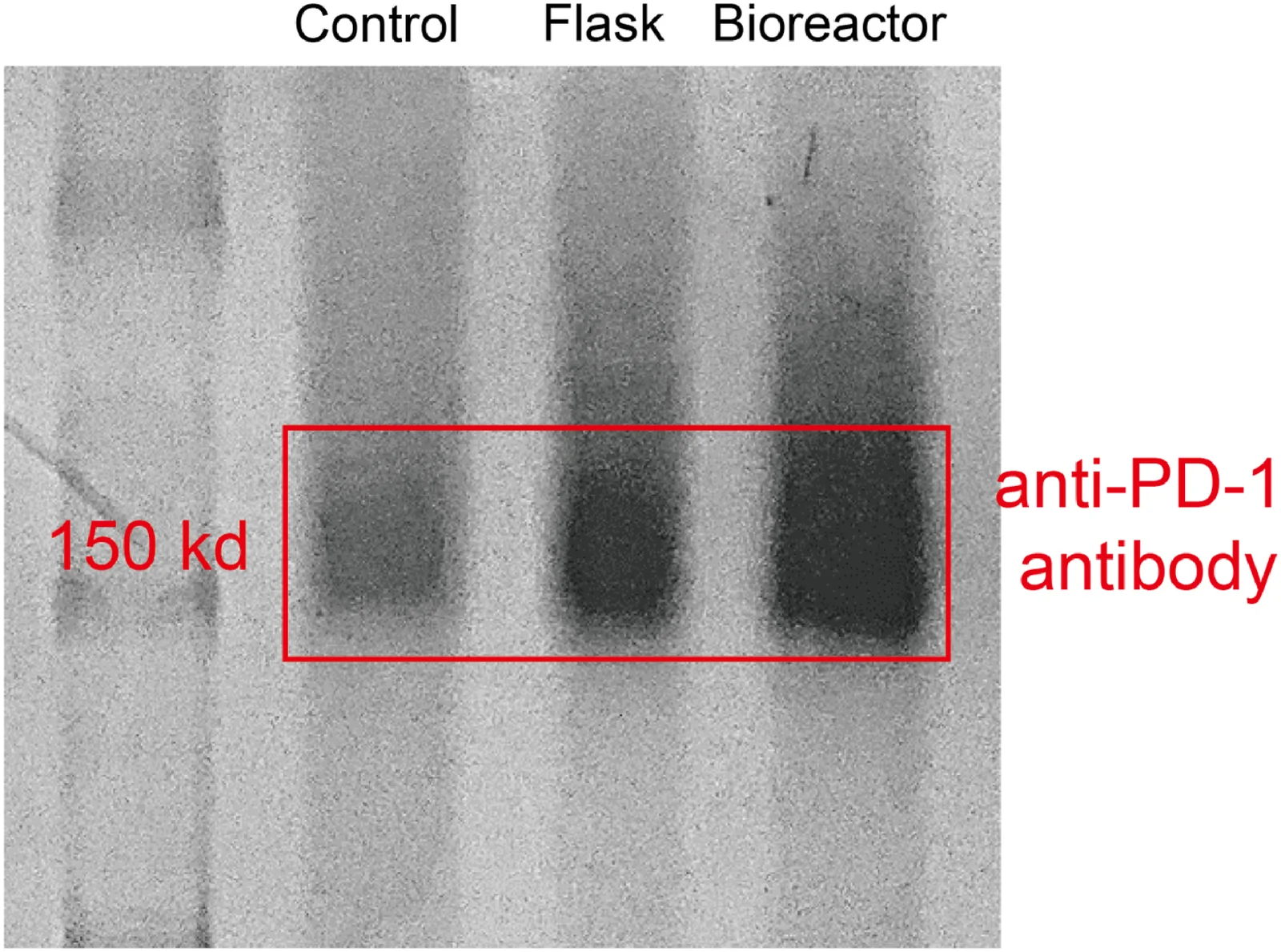
SDS-PAGE of anti-PD-1 antibody expression by Expi293F cells grown in different conditions.

The most dramatic enhancement of transcription was achieved with the histone deacetylase inhibitor VPA. When 3 mM VPA was included in the feed medium, the intracellular eGFP mRNA level at 48 h was 2.8 times higher than in the corresponding culture without VPA (Fig. 10B). This strongly demonstrates VPA's ability to potently enhance the transcriptional activity of exogenous DNA, likely by altering chromatin structure or activating transcription factors.

Ultimately, through the integration of all optimized conditions—including the transcriptional burst afforded by VPA—a breakthrough in anti-PD-1 monoclonal antibody production was achieved in the 3L bioreactor, reaching 171.0 mg/L (Fig. 11), a 10.6-fold improvement over the initial process yield. This result underscores the paramount importance of concurrently optimizing the entire transient expression process, from the initial stage of gene delivery and complex formation through to the subsequent gene expression stages of transcription and translation.

## 4 Discussion

Our data provide preliminary support for the hypothesized biphasic regulation of iron ions in PEI-mediated transfection. However, the most interesting aspect lies in the opposing effects observed at the delivery and expression stages, which we discuss below.

The screening of various metal ions yielded intriguing insights, revealing that their impacts on transfection efficiency are highly specific and not merely a reflection of their general effects on cell viability. Among the tested metal ions, Ca^2+^ showed the most pronounced enhancement of transfection efficiency, consistent with previously reported effects [43]. In stark contrast, the initial inhibitory effect noted for FAC in the screening medium, despite its neutral or slightly positive impact on cell growth, pointed towards a more targeted interference with the transfection mechanism itself [44]. This dissociation between cell growth promotion and transfection inhibition prompted a focused and detailed investigation into the role of iron. Using iron-free CD03 medium, we found that transfection efficiency inversely correlated with iron concentration for all three iron sources tested (Fig. 3), with FAC showing the best performance. The superior performance of FAC over the simpler iron salts is likely attributable to the chelation of iron by citrate, which can modulate its redox activity and bioavailability, potentially reducing the generation of reactive oxygen species and providing a more controlled delivery of iron to the cells. It should be noted that the iron-free CD03 medium lacks transferrin. Consequently, the iron bioavailability from FAC may differ in other media formulations, warranting caution when transferring this protocol.

The most significant mechanistic insight from this work was provided by TEM analysis, which directly linked the iron concentration to the physical state of the PEI/DNA complexes. TEM analysis revealed a clear concentration-dependent morphological transition of PEI/DNA complexes, which directly paralleled the transfection efficiency profile. Previous studies have shown that increased polyplex particle size restricts cellular entry, while optimal condensation facilitates intracellular DNA release. Our TEM observations are consistent with this established relationship. Conversely, at high concentrations, the interference is catastrophic, leading to complexes that are likely too large for efficient endocytosis, too unstable in the extracellular environment, or simply incapable of protecting the DNA payload. This direct evidence underscores that the primary inhibitory effect of high iron concentration is exerted at the very first barrier: the formation of a functional gene delivery vehicle [45].

However, the qPCR data complicated this picture by revealing a contrasting, concentration-dependent enhancement of transgene mRNA by FAC once the DNA had been successfully delivered. Iron is an indispensable cofactor for a plethora of cellular enzymes, including ribonucleotide reductase, various subunits of the mitochondrial electron transport chain, and potentially components of the transcriptional machinery itself [46]. Therefore, within the intracellular milieu, iron facilitates the metabolic and enzymatic processes required for robust transcription and, subsequently, translation [47]. This creates a fascinating dichotomy: iron is a hindrance at the gateway of delivery but a facilitator once the gateway is crossed. This dual mechanism explains why both iron-deficient and iron-overloaded conditions are suboptimal for transient protein production [48].

The potent transcriptional enhancement elicited by VPA further reinforces the importance of the intracellular environment for maximizing recombinant protein yields [49]. As a broad-spectrum histone deacetylase inhibitor, VPA is known to induce chromatin relaxation, thereby increasing the accessibility of exogenous DNA to the transcriptional apparatus [50]. The observed 2.8-fold increase in mRNA levels upon VPA addition powerfully demonstrates that the transcriptional and translational capacities of the host cell, even in a highly transferable line like Expi293F, are not saturated under standard conditions. The synergistic or additive potential of combining optimized metal ion concentrations with epigenetic modulators like VPA opens up promising avenues for pushing transient expression titers to new heights, as spectacularly demonstrated by the 171.0 mg/L antibody yield achieved in the integrated bioreactor run.

The systematic workflow presented here offers a practical strategy for rapidly developing high-yielding transient expression processes. The final titer of 171.0 mg/L ranks among the highest reported for PEI-mediated transient transfection in Expi293F cells, underscoring the translational potential of our approach. However, the present study did not examine potential sub-lethal cellular responses, such as early apoptosis or oxidative stress, that might be induced by high iron concentrations. Future studies may further investigate sub-lethal cellular responses and employ orthogonal analytical methods such as Western blotting for impurity detection. While our findings are robust and mechanistically informative, the generalizability of the optimal iron concentration and the specific morphological effects on PEI/DNA complexes to other cell lines, transfection reagents, or recombinant proteins remains to be established. Furthermore, this study did not examine the impact of iron supplementation on product quality attributes, such as glycosylation patterns or aggregation, which are critical for therapeutic antibody development. These constitute notable limitations that should be addressed in future investigations. In conclusion, the findings presented in this study should be interpreted as preliminary and hypothesis-generating rather than definitive. They are primarily based on observations in a single cell line (Expi293F) and using a limited set of recombinant proteins (eGFP and anti-PD-1). While our data suggest a concentration-dependent dual role of iron ions in PEI-mediated transfection, the generalizability of the optimal iron concentration and the specific morphological effects on PEI/DNA complexes to other cell lines, transfection reagents, or recombinant proteins remains to be established. Further studies are therefore warranted to validate and extend these initial findings.

## 5 Conclusion

In summary, as a preliminary investigation, this study provides initial evidence for establishing a high-efficiency transient transfection platform for Expi293F cells through systematic process optimization and mechanistic investigation. The research revealed a concentration-dependent dual role of iron, which was mechanistically linked to both polyplex stability and transcriptional enhancement. This understanding enabled a 10.6-fold improvement in antibody yield. The integrated optimization strategy, combining refined process parameters, FAC supplementation, and valproic acid-mediated transcriptional enhancement, achieved a remarkable anti-PD-1 antibody yield of 171.0 mg/L in 3L bioreactor, representing a 10.6-fold improvement over initial conditions. These results demonstrate that precise control of iron concentration is a simple and effective strategy to enhance PEI-mediated transient transfection for therapeutic antibody production. In conclusion, this study offers preliminary evidence that precise control of iron concentration is a simple and effective strategy to enhance PEI-mediated transient transfection. However, further studies are warranted to validate the universal applicability of this approach and to systematically evaluate its effects on product quality and process scalability across diverse production systems.

## CRediT authorship contribution statement

**Yu Liu:** Writing – original draft, Visualization, Validation, Methodology, Conceptualization. **Shang Xiao:** Visualization, Methodology, Investigation, Conceptualization. **Yuqian Gui:** Visualization, Methodology, Investigation, Conceptualization. **Senyi Gong:** Methodology, Investigation, Conceptualization. **Yuwen Hu:** Investigation, Formal analysis, Data curation. **Jinzhao He:** Investigation, Formal analysis, Data curation. **Touseef Ur Rehman:** Investigation, Formal analysis, Data curation. **Ali Mohsin:** Writing – review & editing, Project administration, Methodology, Investigation. **Meijin Guo:** Writing – review & editing, Project administration, Methodology, Conceptualization.

## Declaration of generative AI use

During the preparation of this work the authors used Deepseek AI in order to refine grammatical errors and conduct spelling checks. After using this tool, the authors reviewed and edited the content as needed and take full responsibility for the content of the published article.

## Declaration of competing interest

All authors declare that they have no competing interests, whether financial or personal, that could be perceived as influencing this work.

## Data Availability

Data will be made available on request.
