# Supplementary material for: Optimization of PEI-mediated transient transfection for antibody production in Expi293F cells by iron ion supplementation
Source: Biochem Biophys Rep. 2026 Sep 21;48:102799. doi: 10.1016/j.bbrep.2026.102799 (PMC13631826; doi:10.1016/j.bbrep.2026.102799)
Supplement: Supplementary file 1 [file mmc1.docx]

**Title**

**Optimization of PEI-Mediated Transient Transfection for Antibody Production in Expi293F Cells by Iron Ion Supplementation**

**Authors**

Yu Liu^a,1^, Shang Xiao^a,1^, Yuqian Gui^a^, Senyi Gong^a^, Yuwen Hu^a^, Jinzhao He^a^, Touseef Ur Rehman^a^, Ali Mohsin^a^*, Meijin Guo^a^*

**Affiliations**

^a^State Key Laboratory of Bioreactor Engineering, East China University of Science and Technology, Shanghai 200237, China

^1^Yu Liu and Shang Xiao contributed equally to this work.

***Corresponding authors**

**Dr. Ali Mohsin:** Address: P.O. box 329^#^, East China University of Science and Technology, 130 Meilong Rd., Shanghai 200237, P. R. China. Tel: +86 13162094883. E-mail: alimohsin@ecust.edu.cn

**Prof. Meijin Guo:** Address: P.O. box 329#, East China University of Science and Technology, 130 Meilong Rd., Shanghai 200237, P. R. China. Tel: +86 21 64253011; Fax: +86 21 64253702. E-mail: guo_mj@ecust.edu.cn

**Table S1: Summary of amplicon size and annealing temperature of the primers used in this study**

| Primer Name | Sequence (5'→3') | Amplicon Size (bp) | Annealing Temperature (°C) | Length |
| --- | --- | --- | --- | --- |
| eGFP-F | GCATCCCGACTTCTTTAA | ~200 | 60 | 18 |
| eGFP-R | CAGCCGCGTGTTTTCTTC |  |  | 18 |
| β-actin-F | CATTGCTGACAGGATGCAGAAGG | ~150 | 60 | 23 |
| β-actin-R | TGCTGGAAGGTGGACAGTGAGG |  |  | 22 |

**Figure S1.** SDS-PAGE analysis of eGFP protein expression in cell lysates. EGFP protein corresponding to Figure 5B. Sample CD01 represents cell culture using CD01 medium containing iron ions. Sample CD03 represents cell culture using CD03 medium without iron ions. Samples 5, 10, 20, 40, and 80 µ M FAC represent the use of CD03 medium without iron ions and the addition of 5, 10, 20, 40, and 80 µ M FAC for cell culture.

**Figure S2.** SDS-PAGE of anti-PD-1 antibody expression by Expi293F cells grown in different conditions. (A) Anti-PD-1 antibody corresponding to Figure 6B. Samples 10, 20, 40, and 80 µ M FAC represent the use of CD03 medium without iron ions and the addition of 5, 10, 20, 40, and 80 µ M FAC for cell culture. (B) Anti-PD-1 antibody corresponding to Figure 11. Sample Control represents cell culture using CD03 medium. Sample Flask represents cell culture using CD03 medium with the addition of 10 µ M FAC and 3 mM VPA in shake flasks. Sample Bioreactor represents cell culture using CD03 medium with the addition of 10 µ M FAC and 3 mM VPA in Bioreactor.
